# A 4H-SiC CMOS Oscillator-Based Temperature Sensor Operating from 298 K up to 573 K

**DOI:** 10.3390/s23249653

**Published:** 2023-12-06

**Authors:** Nicola Rinaldi, Rosalba Liguori, Alexander May, Chiara Rossi, Mathias Rommel, Alfredo Rubino, Gian Domenico Licciardo, Luigi Di Benedetto

**Affiliations:** 1 Department of Industrial Engineering, University of Salerno, Via Giovanni Paolo II, 132, 84084 Fisciano, SA, Italy; nrinaldi@unisa.it (N.R.); rliguori@unisa.it (R.L.); arubino@unisa.it (A.R.); gdlicciardo@unisa.it (G.D.L.); 2 Fraunhofer Institute for Integrated Systems and Device Technology (IISB), Schottkystraße 10, 91058 Erlangen, Germany; alexander.may@iisb.fraunhofer.de (A.M.); chiara.rossi@iisb.fraunhofer.de (C.R.); mathias.rommel@iisb.fraunhofer.de (M.R.)

**Keywords:** wide band-gap semiconductor, silicon carbide, temperature sensor based on oscillator, process parameter variation

## Abstract

In this paper, we propose a temperature sensor based on a 4H-SiC CMOS oscillator circuit and that is able to operate in the temperature range between 298 K and 573 K. The circuit is developed on Fraunhofer IISB’s 2 μm 4H-SiC CMOS technology and is designed for a bias voltage of 20 V and an oscillation frequency of 90 kHz at room temperature. The possibility to relate the absolute temperature with the oscillation frequency is due to the temperature dependency of the threshold voltage and of the channel mobility of the transistors. An analytical model of the frequency-temperature dependency has been developed and is used as a starting point for the design of the circuit. Once the circuit has been designed, numerical simulations are performed with the Verilog-A BSIM4SiC model, which has been opportunely tuned on Fraunhofer IISB’s 2 μm 4H-SiC CMOS technology, and their results showed almost linear frequency-temperature characteristics with a coefficient of determination that was higher than 0.9681 for all of the bias conditions, whose maximum is 0.9992 at a $V_{DD}$ = 12.5 V. Moreover, we considered the effects of the fabrication process through a Monte Carlo analysis, where we varied the threshold voltage and the channel mobility with different values of the Gaussian distribution variance. For example, at $V_{DD}$ = 20 V, a deviation of 17.4% from the nominal characteristic is obtained for a Gaussian distribution variance of 20%. Finally, we applied the one-point calibration procedure, and temperature errors of +8.8 K and −5.8 K were observed at $V_{DD}$ = 15 V.

## 1. Introduction

4H-polytype silicon carbide semiconductor material is widely used for high temperature applications [1,2,3], and the possibility to fabricate integrated circuits (ICs) allows for an extension of its application fields. Up to now, both unipolar and bipolar 4H-SiC IC technologies have been developed: on bipolar technology, a Bipolar Junction Transistor based on multi-epitaxial stacks is used in analog [4,5,6] and digital [7] ICs, and its performance has been demonstrated up to 873 K. The 4H-SiC Complementary Metal Oxide Semiconductor Field Effect Transistor, CMOS technology proposed by Raytheon, has been developed, and analog and digital building blocks have been fabricated [8], such as, for example, a Positive-To-Absolute-Temperature (PTAT) circuit in the range between 298 K and 573 K, and with a maximum deviation from the ideal linear curve of 33% [9]. Recently, Fraunhofer IISB provided a 4H-SiC 2 μm-CMOS technology [10] and several ICs have been proposed, like CMOS Complementary-To-Absolute-Temperature (CTAT), a sensor in the range between 298 K and 438 K and with a sensitivity of 7.5 mV/K [11], or a temperature sensor based on a p-n diode from 297 K and 873 K, with an $R^{2}=0.9998$ of the voltage-temperature characteristic. Moreover, such technology is also compatible with other device structures that are useful for sensing temperature and ultraviolet radiation [12].

All the 4H-SiC-based proposed temperature sensors transduce temperature in electrical quantities, either through the difference of Gate Source Voltages ($\Delta V_{GS}$) between two MOSFETs [11], or through the difference of diode forward voltages [2,3,9,13,14]. However, 4H-SiC diodes with good performances have vertical structures and they are incompatible with VLSI circuits, whereas, although MOSFETs can be used, they need an integrated circuit in order to read out the voltage-temperature signal. Hence, the study of 4H-SiC CMOS circuits is a relevant topic, both for investigating their potentiality as read-out circuits for sensors and to propose new types of sensors. Among these last, temperature sensors based on time conversion can be a valid, fully compatible, 4H-SiC CMOS alternative, but they have been reported only in Si CMOS technology [15,16]. It is worth noting that the fabrication process quality is still poor because the 4H-SiC CMOS technology is at an early stage, and the variation in the fabrication process parameter on circuit performances is a mandatory analysis.

In this paper, a first all-CMOS temperature sensor based on an oscillator circuit is proposed. It converts the operating temperature into an oscillation frequency of triangular or square waveform voltages. The circuit is fully compatible with the 4H-SiC 2 μm-CMOS technology and its performances are extracted from numerical simulation up to an operating temperature of 573 K. Considering that the oscillation frequency is related to the charge and discharge of an integrated capacitance through the device currents, we can obtain a temperature-frequency conversion because those currents have a temperature dependency. The paper is organized as follows: in Section 2, the topology, the operating principle, and the design of the sensor are reported; in Section 3, numerical simulation results are shown, focusing both on the differences from the analytical design due to second-order device phenomena and on the effects of the fabrication process variations; Section 4 and Section 5 are, respectively, the layout and the conclusions.

## 2. The Topology of the Temperature-Sensing Oscillator

**Figure 1:**
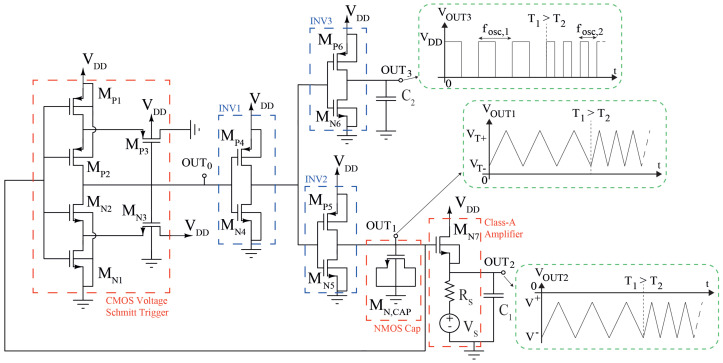
The topology of the proposed temperature sensor, based on an oscillator circuit.

The circuit of Figure 1 is the proposed sensor and is based on an oscillator whose frequency is uniquely related to the temperature. In the following, we report the single sub-circuits and the design of the circuit.

The circuit is an astable multivibrator and it is composed of a CMOS Schmitt trigger, three CMOS inverters, a n-type MOS capacitor, and a common drain NMOSFET amplifier. The circuit can generate a square waveform voltage, $OUT_{3}$, thanks to the CMOS inverter $INV_{3}$, which is opportunely sized to load capacitance $C_{2}$, and a triangular waveform voltage at the $OUT_{1}$. The waveforms of $OUT_{1}$ and $OUT_{3}$ are related to each other thanks to the integrator composed of $INV_{2}$ and NMOS Cap. The sensing circuit is biased to positive voltage, $V_{DD}$, and a negative voltage, $V_{S}$, which needs to bias the Class-A amplifier.

### 2.1. CMOS Voltage Schmitt Trigger

**Figure 2:**
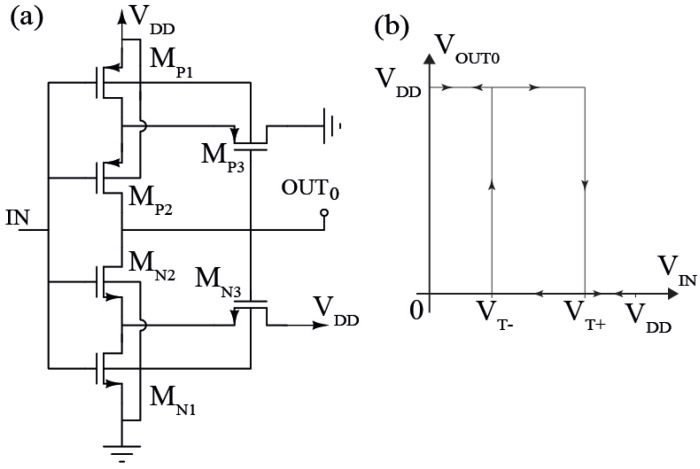
(**a**) CMOS voltage Schmitt trigger schematic and (**b**) its transcharacteristic.

The 4H-SiC CMOS voltage Schmitt trigger schematic and transcharacteristic are reported, respectively, in Figure 2a,b [17,18]. It has three pairs of CMOS transistors, which are $M_{N1-3}$ and $M_{P1-3}$, and the input and output signals are, respectively, IN and $OUT_{0}$.

Until $V_{IN}$ is lower than $V_{TH_{N_{1}}}$, which is the threshold voltage of the NMOSFET $M_{N_{1}}$, the stacked PMOS transistors $M_{P_{1}}$ and $M_{P_{2}}$ are in linear operation region, and the source follower $M_{N_{3}}$ is in saturation operation mode, whereas $M_{P_{3}}$, $M_{N_{1}}$, and $M_{N_{2}}$ are turned off. The drain potential of the $M_{N_{1}}$ is $V_{DD}-V_{TH_{N_{3}}}$ until $V_{IN}$ remains lower than $V_{TH_{N_{1}}}$. When $V_{IN}\geq V_{TH_{N_{1}}}$, $M_{N_{1}}$ is turned on and biased in the saturation region. When $V_{IN}$ is higher than $V_{DS_{N_{1}}}+V_{TH_{N_{2}}}$, the transistor $M_{N_{2}}$ turns on so that the positive feedback loop, composed of $M_{N_{2}}$ and $M_{N_{3}}$, quickly drops the $OUT_{0}$ voltage to ground [19]. Meanwhile, the source follower $M_{P_{3}}$ turns on and brings the source potential of $M_{P_{2}}$ to a low value so that this last one turns off. The high threshold voltage, $V_{T+}$, can be calculated as follows [17]:

(1) $$V_{IN}=V_{T^{+}}=\frac{V_{DD}-\left|V_{TH_{P}}\right|+\sqrt{\frac{K_{N}}{K_{P}}}V_{TH_{N}}}{1+\sqrt{\frac{K_{N}}{K_{P}}}}+\frac{\sqrt{\frac{K_{N}}{K_{P}}}\left(V_{DD}-V_{TH_{N}}\right)}{\left(1+\sqrt{\frac{W_{N_{1}}L_{N_{3}}}{W_{N_{3}}L_{N_{1}}}}\right)\left(1+\sqrt{\frac{K_{N}}{K_{P}}}\right)}$$

where $K_{N}=K_{N_{1}}//K_{N_{2}}$ and $K_{P}=K_{P_{1}}//K_{P_{2}}$, $K_{Ni}=K_{N}W_{Ni}/L_{Ni}$, $K_{Pi}=K_{P}W_{Pi}/L_{Pi}$. Also, $K_{N}=\mu_{N}C_{OX}$ and $K_{P}=\mu_{P}C_{OX}$ are, respectively, the transconductance coefficients of the NMOSFET and PMOSFET, $\mu_{N(P)}$ is the carrier channel mobility, $C_{OX}$ is the gate oxide capacitance, and $W_{i}$ and $L_{i}$ are, respectively, the width and the length of the *i*-transistor. Moreover, at $V_{IN}=V_{T+}$ and before the positive feedback loop intervenes, it is reasonable that $V_{OUT0}=V_{DD}$ and that the following relation is valid [19]:

(2) $$\frac{K_{N_{1}}}{K_{N_{3}}}={\left(\frac{V_{DD}-V_{T^{+}}}{V_{T^{+}}-V_{TH_{N}}}\right)}^{2}$$

In the reverse direction, i.e., where $V_{IN}$ reduces from $V_{DD}$ to ground, the symmetrical process takes place. At $V_{IN}=V_{DD}$, the transistors $M_{N_{1}}$, $M_{N_{2}}$, and $M_{P_{3}}$ conduct, while $M_{N_{3}}$, $M_{P_{1}}$, and $M_{P_{2}}$ are turned off. Then, at $V_{IN}\leq V_{DD}-\left|V_{TH_{P}}\right|$, the transistor $M_{P_{1}}$ conducts, and when $V_{IN}$ becomes lower than $V_{DD}-V_{SD_{P_{1}}}-\left|V_{TH_{P_{2}}}\right|$, the transistor $M_{P_{2}}$ turns on and the positive feedback loop, composed by $M_{P_{2}}$ and $M_{P_{3}}$, is closed and brings the output voltage to $V_{DD}$. Simultaneously, the source follower $M_{N_{3}}$ turns on and increases the source potential of $M_{N_{2}}$, inducing its turn-off. The low threshold voltage, $V_{T-}$, can be calculated using the following equation [17]:

(3) $$V_{IN}=V_{T^{-}}=\frac{V_{DD}-\left|V_{TH_{P}}\right|+\sqrt{\frac{K_{N}}{K_{P}}}V_{TH_{N}}}{1+\sqrt{\frac{K_{N}}{K_{P}}}}-\frac{V_{DD}-\left|V_{TH_{P}}\right|}{\left(1+\sqrt{\frac{W_{P_{1}}L_{P_{3}}}{W_{P_{3}}L_{P_{1}}}}\right)\left(1+\sqrt{\frac{K_{N}}{K_{P}}}\right)}$$

Moreover, at $V_{IN}=V_{T-}$ and before the positive feedback loop intervenes, it is reasonable that $V_{OUT0}=0$ and the following relation is valid [19]:

(4) $$\frac{K_{P_{1}}}{K_{P_{3}}}={\left(\frac{V_{T^{-}}}{V_{DD}-V_{T^{-}}-\left|V_{TH_{P}}\right|}\right)}^{2}$$

By defining the values of $V_{DD}$, $V_{T^{+}}$, and $V_{T^{-}}$, one can design the channel sizes of $M_{N1}$ and $M_{N3}$ from (2), and of $M_{P1}$ and $M_{P3}$ from (4); instead, $M_{N2}$ and $M_{P2}$ are obtained, respectively, from (1) and (3).

In the proposed circuit of Figure 1, the $OUT_{0}$ is followed by a CMOS inverter, $INV_{1}$, composed of $M_{N_{4}}$-$M_{P_{4}}$, in order to invert the transcharacteristic and to make it compatible with the second part of the circuit. Moreover, $INV_{1}$ drives the loads connected to the Schmitt trigger so that the dynamic behavior is preserved, as shown in the Section 2.2, Section 2.3 and Section 2.4.

### 2.2. Integrator and Output Stages

The NMOS capacitor, $M_{N,CAP}$, of Figure 1, is an NMOS-based capacitor and it has been preferred to MIM-caps due to the lack of a Verilog-A model of this last in our actual 4H-SiC CMOS technology. Its gate is controlled by the output of the Schmitt trigger and is driven by the CMOS inverter $INV_{2}$, composed by $M_{N_{5}}$- $M_{P_{5}}$, with a constant current so that a triangular waveform appears at the input of the Schmitt trigger, i.e., $V_{OUT1}$.

The ramp time is defined by the charging and discharging of $M_{N_{CAP}}$: when $V_{G_{N,CAP}}$ increases toward $V_{DD}$, starting from $V_{T^{-}}$, the time constant is $\tau_{P}=R_{P}C_{L}$ and the charging stops at $V_{G_{N,CAP}}=V_{T^{+}}$ because the Schmitt trigger changes state; when $V_{G_{N,CAP}}$ decreases toward ground starting from $V_{T^{+}}$, the time constant is $\tau_{n}=R_{N}C_{L}$ and the discharging stops at $V_{G_{N,CAP}}=V_{T^{-}}$. The capacitance $C_{L_{I}}$ is the total load capacitance at the $OUT_{1}$ terminal and the equivalent resistances of the time constants are [20]:

(5a) $$\begin{array}{cc} R_{N} & =\frac{L_{N}}{\mu_{N}C_{OX}W_{N}\left(V_{DD}-V_{TH_{N}}\right)} \end{array}$$

(5b) $$\begin{array}{cc} R_{P} & =\frac{L_{P}}{\mu_{P}C_{OX}W_{P}(V_{DD}-|V_{TH_{P}}\left|\right)} \end{array}$$

Moreover, to avoid frequency and waveform distortions, we added two output stages needed to charge the probe capacitances $C_{1}$ and $C_{2}$, which are of the order of tens of pF. Observing the circuit of the Figure 1, one is a Class-A amplifier based on NMOS $M_{N_{7}}$ for the triangular waveform output, $V_{OUT2}$, and the other is a CMOS inverter $INV_{3}$, composed of $M_{N_{6}}$-$M_{P_{6}}$, for the square waveform output, $V_{OUT3}$. The $V_{OUT2}$ is the same waveform of $V_{OUT1}$ with a level-shift voltage of $V_{GS_{N7}}$, and hence, the maximum and minimum voltages of $V_{OUT2}$ are $V_{+}=V_{T+}-V_{GS}^{+}$ and $V_{-}=V_{T-}-V_{GS_{7}}^{+}$, respectively, where $V_{GS7}^{+}$ and $V_{GS7}^{-}$ are the $V_{GS_{N7}}$ when $V_{G7}$ is equal, respectively, to the $V_{T^{+}}$ and $V_{T^{-}}$ values. Moreover, upon observing Figure 1, the Class-A amplifier is biased by a resistor, $R_{S}$ and a bias voltage, $V_{S}$, which are externally applied to the $OUT_{2}$ terminal.

### 2.3. Evaluation of the Oscillation Frequency

The oscillation frequency, $f_{OSC}$, can be calculated considering the charge and discharge times of $M_{N_{CAP}}$, $t_{CAP}$, together with the time propagation delays of the Schmitt trigger and of $INV_{1}$. Indeed, one obtains:

(6) $$f_{OSC}=\frac{1}{2t_{CAP}+t_{pHL_{trigger}}+t_{pLH_{trigger}}+t_{pHL_{INV_{1}}}+t_{pLH_{INV_{1}}}}$$

where $t_{pHL{\left(LH\right)}_{trigger}}$ and $t_{pHL{\left(LH\right)}_{INV_{1}}}$ are the time propagation delays high-low (low-high) of the Schmitt trigger and of $INV_{1}$, respectively, and $t_{CAP}$ is the mean time of the charge and discharge time of $M_{N_{CAP}}$. The latter has the following relation in order to design the circuit:

(7) $$t_{CAP}=t_{charge}=t_{discharge}$$

Therefore, we obtain the same time to charge and discharge $M_{N,CAP}$, which means a saw-tooth waveform signal for $V_{OUT1}$ as well as for $V_{OUT2}$, and a duty cycle of 50% for the square waveform of $V_{OUT3}$. To calculate $f_{OSC}$, the single terms of (6) are evaluated as follows [20]:

(8a) $$\begin{array}{cc} t_{charge} & =R_{P_{5}}C_{L_{I}}ln\left(\frac{V_{DD}-V_{T^{-}}}{V_{DD}-V_{T^{+}}}\right) \end{array}$$

(8b) $$\begin{array}{cc} t_{discharge} & =R_{N_{5}}C_{L_{I}}ln\left(\frac{V_{T^{+}}}{V_{T^{-}}}\right) \end{array}$$

(9a) $$\begin{array}{cc} t_{p_{HL_{trigger}}} & =0.69\left(R_{N_{1}}+R_{N_{2}}\right)C_{L_{II}} \end{array}$$

(9b) $$\begin{array}{cc} t_{p_{LH_{trigger}}} & =0.69\left(R_{P_{1}}+R_{P_{2}}\right)C_{L_{II}} \end{array}$$

(10a) $$\begin{array}{cc} t_{p_{HL_{INV_{1}}}} & =0.69R_{N_{4}}C_{L_{III}} \end{array}$$

(10b) $$\begin{array}{cc} t_{p_{LH_{INV_{1}}}} & =0.69R_{P_{4}}C_{L_{III}} \end{array}$$

where the capacitances are as follows:

(11a) $$\begin{array}{cc} C_{L_{I}} & =C_{N_{CAP}}+2C_{GD_{N_{5}}}+2C_{GD_{P_{5}}}+C_{DB_{N_{5}}}+C_{DB_{P_{5}}}+C_{G_{N_{1}}}+C_{G_{N_{2}}}+C_{G_{P_{1}}}+C_{G_{P_{2}}}+C_{G_{N_{7}}} \end{array}$$

(11b) $$\begin{array}{cc} C_{L_{II}} & =C_{G_{N_{4}}}+C_{G_{P_{4}}}+2C_{GD_{N_{2}}}+2C_{GD_{P_{2}}}+C_{DB_{N_{2}}}+C_{DB_{P_{2}}}+C_{G_{N_{3}}}+C_{G_{P_{3}}} \end{array}$$

(11c) $$\begin{array}{cc} C_{L_{III}} & =C_{G_{N_{5}}}+C_{G_{P_{5}}}+C_{G_{N_{6}}}+C_{G_{P_{6}}}+2C_{GD_{N_{4}}}+2C_{GD_{P_{4}}}+C_{DB_{N_{4}}}+C_{DB_{P_{4}}} \end{array}$$

where $C_{G}$, $C_{GD}$, and $C_{DB}$ are, respectively, the Gate, the Gate-Drain, and the Drain-Body capacitance.

The design can be simplified, assuming the following conditions:

- for $C_{N_{CAP}}$ that is higher than other capacitances, one has $C_{L_{I}}≃C_{N_{CAP}}$;
- for $t_{CAP}$ that is higher than the propagation delays, one obtains $f_{OSC}≃0.5t_{CAP}^{-1}$.

About the $OUT_{3}$ signal, $INV_{3}$ loads the probe capacitance $C_{2}$ and the time propagation delay is evaluated as follows:

(12a) $$\begin{array}{cc} t_{p_{HL_{INV_{3}}}} & =0.69R_{N_{6}}C_{2} \end{array}$$

(12b) $$\begin{array}{cc} t_{p_{LH_{INV_{3}}}} & =0.69R_{P_{6}}C_{2} \end{array}$$

Therefore, assuming that all the channel lengths are equal to the minimum one, and that the time propagation delays are negligible for $t_{pHL(LH)}=10^{-3}t_{CAP}$, the design is completed in this way:

- fixing $f_{OSC}$, one has $t_{CAP}=t_{charge}=t_{discharge}=0.5f_{OSC}^{-1}$;
- fixing $C_{N_{CAP}}$, one obtains $W_{P5}$ from (8a) and $W_{N5}$ from (8b);
- fixing $t_{p_{INV_{3}}}$ and $C_{2}$, the channel widths of $INV_{3}$ are calculated from (12);
- fixing $t_{p_{INV_{1}}}$, the channel widths of $INV_{1}$ are calculated from (10);
- fixing $t_{p_{trigger}}$, (9) complete the equations used together with (1)–(4) to design the Schmitt trigger.

Finally, because the $M_{N_{7}}$ class-A output stage loads the probe capacitance $C_{1}$ in order that the triangular waveform is undistorted, its current has to respect the following relation (see Figure 1):

(13) $$I_{C_{3}}=C_{1}\frac{\Delta V}{\Delta t}=C_{1}\frac{V_{+}-V_{-}}{t_{CAP}}$$

Also, considering that $M_{N7}$ is in saturation operation mode and for a selected value of $V_{S}$, it is possible to write:

(14) $$\begin{array}{cc} \{ & \sqrt{\frac{2\left(\frac{V_{+}-V_{S}}{R_{S}}-I_{C_{3}}\right)}{\mu_{N}C_{ox}\frac{W_{7}}{L_{7}}}}+V_{TH_{N}}-V_{GS7}^{+}=0\;\sqrt{\frac{2\left(\frac{V_{-}-V_{S}}{R_{S}}-I_{C_{3}}\right)}{\mu_{N}C_{ox}\frac{W_{7}}{L_{7}}}}+V_{TH_{N}}-V_{GS7}^{-}=0 \end{array}$$

Hence, the values of $W_{N7}$, $L_{N7}$, and $R_{s}$ can be found from (13)–(14).

### 2.4. Design of the Circuit

The equations from (1) to (14) are used to design our circuit with the project specifications of Table 1. The values are from a trade-off among the expected performances of the 4H-SiC CMOS technology and the possibility to make our sensor compatible with the current electronic and the occupied wafer area. The device physical parameters used in (1)–(14) are the threshold voltages of the MOSFETs, $V_{TH_{N}}$ = 5.8 V and $V_{TH_{P}}=-8$ V, the channel mobility, $\mu_{N}$ = 17.14 cm${}^{2}$V${}^{-1}$s${}^{-1}$ and $\mu_{P}$ = 3.52 cm${}^{2}$V${}^{-1}$s${}^{-1}$, and the oxide capacitance, $C_{OX}$ = 62.78 nFcm${}^{-2}$. In the Appendix A, the device physical parameters, characteristics, and extraction procedure are reported.

By using the results of the previous subsections, the design of the proposed circuit is reported in Table 2. The proposed procedure is based on an analytical approach and allows for the definition of a first-order design of the circuit, which can be used as a starting point for the numerical simulations. Indeed, a tuning procedure is required to achieve the project specifications, as has been shown in the following section, due to second-order effects of the transistors, like $4H-SiC/SiO_{2}$ interface defects or fabrication process non-uniformity.

## 3. Numerical Simulation Results and Process Variability

Once the design has been completed, numerical simulations have been performed in the Cadence Virtuoso environment [21] by using a Verilog-A BSIM MOSFETs model [22] whose parameters are opportunely tuned to fit the experimental curves of the 4H-SiC MOSFETs in the temperature range from 298 K to 573 K. The model has been developed by Fraunhofer IISB, and a more detailed description of the 4H-SiC MOSFETs is reported in Appendix A.

**Figure 3:**
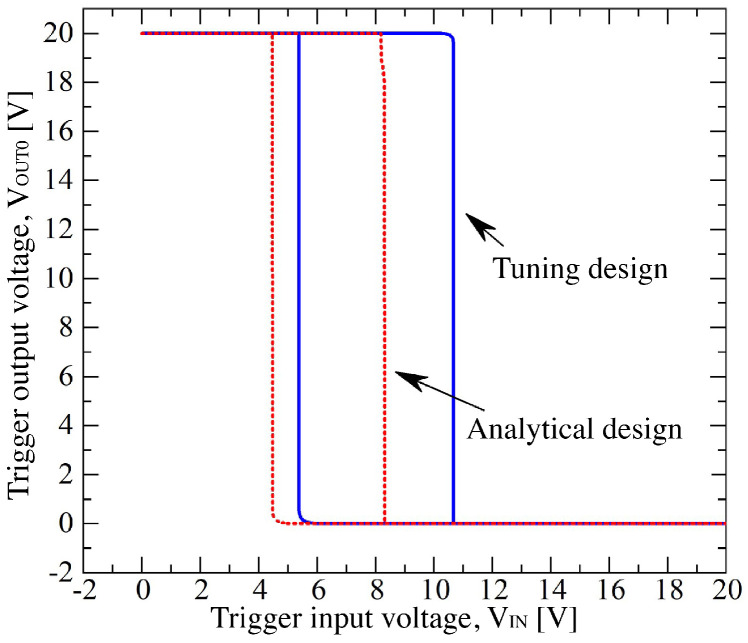
Numerically simulated trans-characteristics of the Schmitt trigger from analytical and tuning designs at T=298 K.

**Figure A3:**
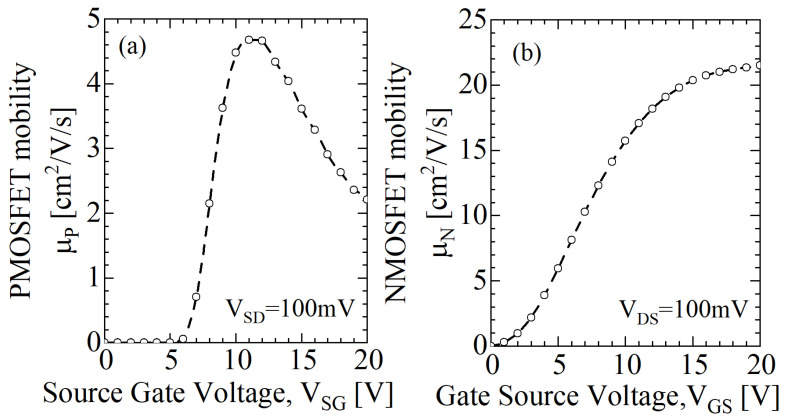
Extracted mobility from the transcharacteristics of Figure A1 by using (A1) for 4H-SiC. (**a**) PMOSFET with $W_{P}/L_{P}=100$ μm/10 μm and (**b**) NMOSFET with $W_{P}/L_{P}=10$ μm/10 μm at $|V_{DS}|=0.1$ V, $|V_{BS}|=0$ V, and at r.t.

Numerical results report some inconsistencies with the project specifications; for example, the oscillation frequency is 85.94 kHz instead of 90 kHz, or the trigger threshold voltages are $V_{T^{+}}=8.14$ V and $V_{T^{-}}=4.4$ V compared to 10 V and 5 V, respectively, as reported in Table 1. Keeping the same $L_{N}$ and $L_{P}$ of Table 2 but varying $W_{N}$ and $W_{P}$, we obtained the design reported in Table 3, and the circuit results show $f_{OSC}=93.8$ kHz, $V_{T^{+}}=10.66$ V, and $V_{T^{-}}=5.37$ V. Indeed, in Figure 3, the comparison between the two designs shows how the asymmetry of the trans-characteristics obtained using the analytical design approach, disappears in the one resulting from the tuning of the transistor sizes. Such differences can be explained by second-order effects, which are neglected in the simplified current model used to extract (1)–(14): for example, they are the saturation of the carrier velocity, the channel length modulation, the bias effect of the body, and the effects of defects at the $4H-SiC/SiO_{2}$ interface on the MOSFET electric behavior [23]. For example, observing Figure A3b, the high density of interface defects modifies the NMOSFET channel mobility dependency on $V_{GS}$ compared to the typical shape in Silicon technology: indeed, in Silicon technology, we expect a step-like curve at $V_{GS}≃V_{TH_{N}}$, and then, a slight decay of $\mu_{N}$ for $V_{GS}>V_{TH_{N}}$ [24]; instead, in 4H-SiC technology, the mobility has a continuous increase with $V_{GS}$, and for $V_{GS}>15$ V, it remains constant.

**Figure 4:**
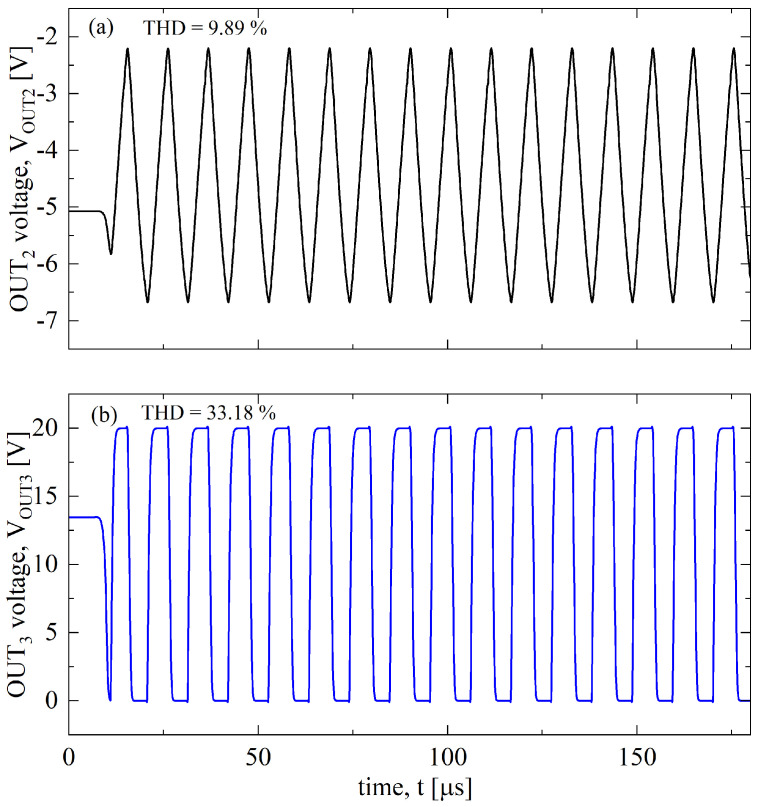
Waveform signals of (**a**) $V_{OUT2}$ and (**b**) of $V_{OUT3}$ at T=298 K.

As our temperature sensor is an oscillator, the total harmonic distortions, THDs, of $V_{OUT2}$ and $V_{OUT3}$ for Table 3 have been evaluated. Indeed, our sensor can be followed by a read-out circuit, like a frequency counter or microcontroller, whose aim is to measure the frequency; such a measurement is as accurate as the waveform is not distorted. These respectively show triangular and square waveforms in Figure 4 at T=298 K, with a THD, respectively, of 9.89% and of 33.18%, which are slightly lower than the pure symmetric waveforms, i.e., THD=12.1% for the triangular and THD=48.3% for the squared waveforms [25].

**Figure 5:**
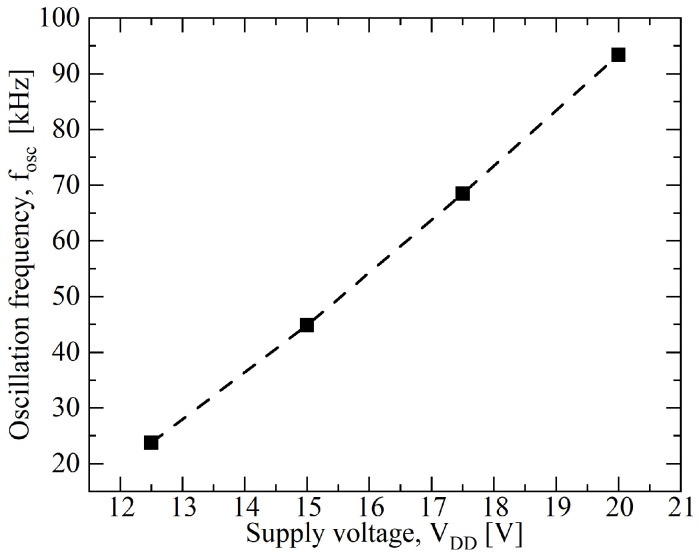
Oscillation frequency, $f_{OSC}$, dependency on power supply voltage, $V_{DD}$, at T=298 K, showing a sensitivity of 9.28 kHz/V.

Moreover, an estimation of the total average power dissipation gives a value of 2.45 mW at 298 K and the bias-frequency sensitivity is 9.28 kHz/V, as shown in Figure 5.

In the following, the effects on the oscillation frequency of the temperature and of the fabrication process variations are analyzed and investigated for the design reported in Table 3.

### 3.1. Oscillation Frequency Dependency on the Temperature

**Figure 6:**
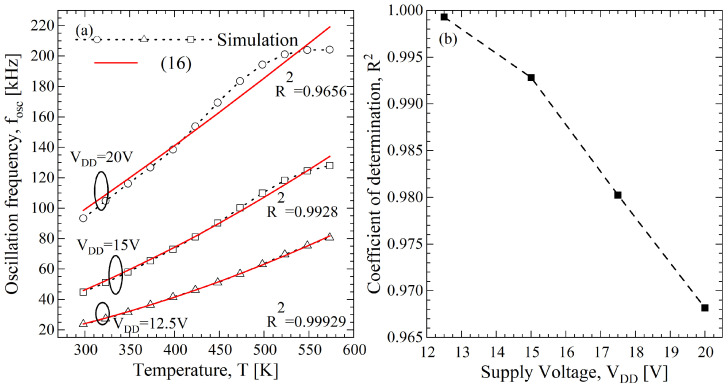
(**a**) Comparisons of the $f_{OSC}$-*T* curves between numerical simulation results, obtained from the Verilog-A BSIM MOSFETs model and the analytical model of (16) at $V_{DD}=12.5$ V and 20 V. (**b**) $R^{2}-V_{DD}$ curve obtained from the $f_{OSC}$-*T* curves.

The oscillation frequency dependency on the temperature, $f_{OSC}$-*T*, can be shown, beginning with the following relation:

(15) $$f_{OSC}≃\frac{1}{t_{charge}+t_{discharge}}=\frac{1}{C_{L_{I}}\left[R_{P_{5}}ln\left(\frac{V_{DD}-V_{T^{-}}}{V_{DD}-V_{T^{+}}}\right)+R_{N_{5}}ln\left(\frac{V_{T^{+}}}{V_{T^{-}}}\right)\right]}$$

where it is assumed that $t_{CAP}\gg \left(t_{CMOS_{INV_{I}}}+t_{trigger}\right)$ in (6). The dependence of the MOSFETs on the temperature is described through the channel mobilities and the threshold voltages, which are explicitly reported in the Appendix A. However, for a first analysis of (15), only the channel mobility is considered because $V_{TH_{N}}$ and $V_{TH_{P}}$ (*i*) appear in $V_{T-}$ and $V_{T+}$, which are divided by $V_{DD}$ and they are also the arguments of a logarithm function; and (*ii*) are in $R_{P_{5}}$ and $R_{N_{5}}$, which are divided by $V_{DD}$. Hence, one obtains:

(16) $$f_{OSC}=\frac{1}{C_{L_{I}}}\frac{1}{\frac{ln\left(\frac{V_{DD}-V_{T^{-}}}{V_{DD}+V_{T^{+}}}\right)}{\mu_{P}\left(T_{0}\right)C_{ox}\frac{W_{P_{5}}}{L_{P_{5}}}V_{DD}\left(1-\frac{|V_{TP}|}{V_{DD}}\right)}+\frac{ln\left(\frac{V_{T^{+}}}{V_{T^{-}}}\right)}{\mu_{N}\left(T_{0}\right)C_{ox}\frac{W_{N_{5}}}{L_{N_{5}}}V_{DD}\left(1-\frac{V_{TN}}{V_{DD}}\right)}}{\left(\frac{T}{T_{0}}\right)}^{\alpha }=A{\left(\frac{T}{T_{0}}\right)}^{\alpha }$$

where *A* includes all the parameters related to the fabrication process and is independent from the temperature, in a first approximation. In Figure 6a, the resulting $f_{OSC}$-*T* curve at $V_{DD}=20$ V is reported and the best fitting of (16) gives A=98.66 kHz and α=1.22 with an $R^{2}=0.9656$. We also investigated the effects of $V_{DD}$ on the linearity of the $f_{OSC}$-*T* curve, varying from 12.5 V to 20 V, and an improvement is obtained at $V_{DD}=12.5$ V with an $R^{2}=0.9992$, as shown in Figure 6b. The $f_{OSC}$-*T* curve at $V_{DD}=12.5$ V is reported in Figure 6a and it is compared with the model having A=23.88 kHz and α=1.883, where a better fitting is evident; however, as is expected from (16), there is a reduction of the value of $f_{OSC}$ from 93.8 kHz to 23.88 kHz, evaluated at T=298 K. For completeness in Figure 6a, we report the $f_{OSC}$-*T* characteristic for the bias voltage of 15 V and we obtain $R^{2}=0.9928$, A=45.92 kHz, and α=1.64.

### 3.2. Effects of Process Parameters Variation

Fabrication process variations are expected, and for example, in 4H-SiC CMOS technology, they can be related to variations on the activation process of the Aluminum p-type doping atoms [26], on the uniformity of the doping concentration and of the oxide thickness, or on the quality of the contact resistance of doped regions [27]. All these fabrication process variations can be modeled, in a first analysis, on variations in the channel mobility and of the threshold voltage, and then used for Monte Carlo analysis to assess the sensitivity of the circuit. In particular, the analysis is a 1000 point process Monte Carlo and consists of evaluating the $f_{OSC}$-*T* curves, considering a Gaussian distribution for $\mu_{N(P)}$ and $V_{TH,N(P)}$, which is defined by a standard deviation (σ) and a mean value (μ) through the following distribution:

(17) $$p\left(x\right)=\frac{1}{\sigma \sqrt{2\pi }}e^{-\frac{{\left(x-\mu \right)}^{2}}{2\sigma^{2}}}$$

During the analysis, we varied the supply voltage from 12.5 V to 20 V and the ratio σ/μ of the Gaussian distribution from ±10% to ±20%, either for both parameters or singularly. To compare the cases, we use the oscillation frequency variation, $f_{OSC,var}$, defined as follows:

(18) $$f_{OSC,var}\left(T\right)=\frac{|f_{OSC,\sigma }\left(T\right)-f_{OSC}\left(T\right)|}{f_{OSC}\left(T\right)}100$$

**Figure 7:**
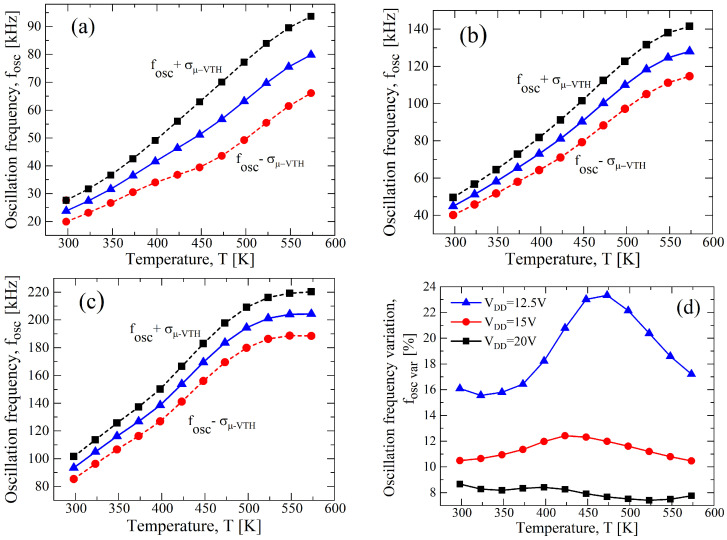
Results of a 1000 points process Monte Carlo analysis for (**a**) $V_{DD}=12.5$ V, (**b**) $V_{DD}=15$ V, and (**c**) $V_{DD}=20$ V, with σ/μ=±0.1 for $V_{TH,N(P)}$ and for $\mu_{N(P)}$. (**d**) $f_{OSC,var}$ as function of the temperature in terms of $V_{DD}$ resulting from (**a**–**c**).

In Figure 7a–c, the $f_{OSC}$-*T* curves for a σ/μ=±0.1 and at different values of $V_{DD}$ are reported. Although the curve of Figure 7a for the case $V_{DD}=12.5$ V shows a better linearity, a higher $f_{OSC,var}$ appears: indeed, it is almost 23%, and in Figure 7d, the $f_{OSC,var}$-*T* curve is shown. Instead, observing Figure 7c for $V_{DD}=20$ V, a maximum $f_{OSC,var}$ of 8% is achieved, but a worse linearity is obtained, as shown in Figure 6. Hence, a supply voltage of 15 V allows for a good trade-off between the process parameter variation and the linearity, as is clearly shown in Figure 7b,d.

**Figure 8:**
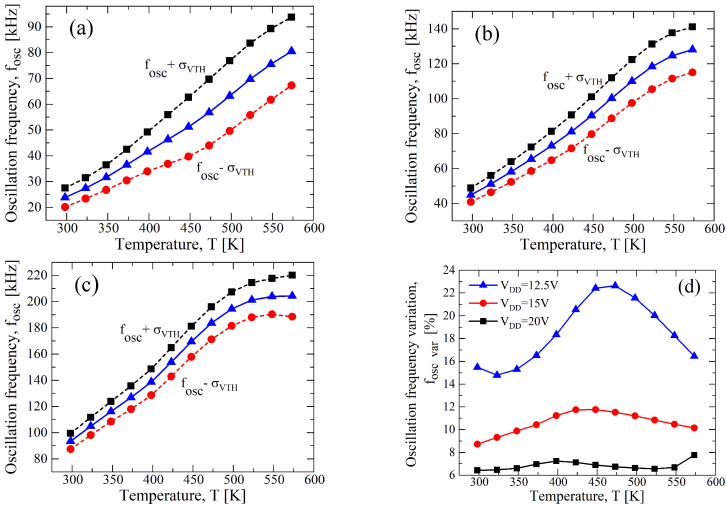
Results of a 1000 points process Monte Carlo analysis for (**a**) $V_{DD}=12.5$ V, (**b**) $V_{DD}=15$ V, and (**c**) $V_{DD}=20$ V, with σ/μ=±0.1 for only $V_{TH,N(P)}$, whereas $\mu_{N(P)}$ is constant at nominal value of Table 1. (**d**) $f_{OSC,var}$ as function of the temperature in terms of $V_{DD}$ resulting from (**a**–**c**).

**Figure 9:**
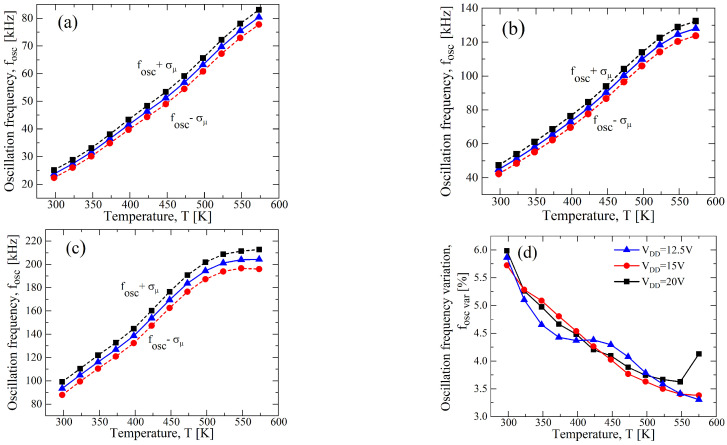
Results of a 1000 points process Monte Carlo analysis for (**a**) $V_{DD}=12.5$ V, (**b**) $V_{DD}=15$ V, and (**c**) $V_{DD}=20$ V, with σ/μ=±0.1 for only $\mu_{N(P)}$, whereas $V_{TH,N(P)}$ is constant at nominal value of Table 1. (**d**) $f_{OSC,var}$ as function of the temperature in terms of $V_{DD}$ resulting from (**a**–**c**).

To understand the effects of the single device parameter on the performance of the circuit, we performed Monte Carlo analysis by singularly varying either $V_{TH,N(P)}$ or $\mu_{N(P)}$. In Figure 8, a σ/μ of ±10% for $V_{TH,N(P)}$ with a constant value of $\mu_{N(P)}$ at the nominal value reported in Table 1 shows a stronger variation in the oscillation frequency as a function of the temperature than the case of a σ/μ of ±10% for $\mu_{N(P)}$ with a constant value of $V_{TH,N(P)}$, as reported in Figure 9. Indeed, observing Figure 8d, the maximum $f_{OSC,var}$ related to the variations in $V_{TH,N(P)}$ are 22.64%, 11.76%, and 7.24% for $V_{DD}$, equal to 12.5 V, 15 V, and 20 V, respectively, whereas for the variations in $\mu_{N(P)}$, they are around 5.8% for all bias conditions (see Figure 9d). That is also confirmed, considering that the maximum variation for the cases of Figure 7 is almost similar to that of Figure 8, and in Table 4, they are reported for ease of reading. However, the best trade-off in terms of the linearity and maximum variation is still at $V_{DD}=15$ V for both cases.

**Figure 10:**
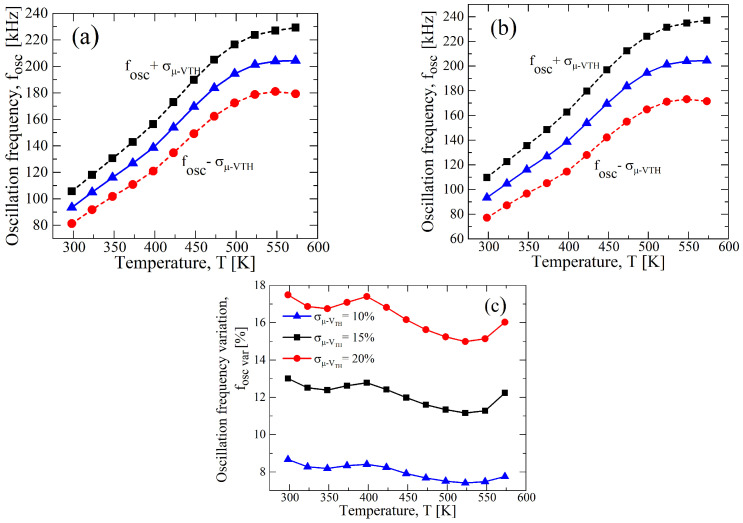
Results of a 1000 points process Monte Carlo analysis for (**a**) σ/μ=±0.15, (**b**) σ/μ=±0.2 for $\mu_{N(P)}$ and $V_{TH,N(P)}$ at $V_{DD}=20$ V. (**c**) $f_{OSC,var}$ as function of the temperature for different values of σ of $\mu_{N(P)}$ and $V_{TH,N(P)}$ at $V_{DD}=20$ V.

**Figure 11:**
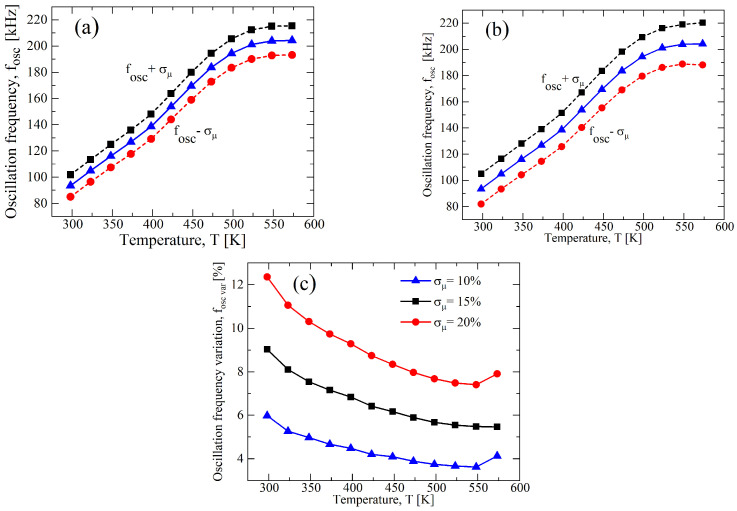
Results of a 1000 points process Monte Carlo analysis for (**a**) σ/μ=±0.15 and (**b**) σ/μ=±0.2, for only $\mu_{N(P)}$, whereas $V_{TH,N(P)}$ is constant at nominal value of Table 1 at $V_{DD}=20$ V. (**c**) $f_{OSC,var}$ as function of the temperature for different values of σ of $\mu_{N(P)}$ with $V_{TH,N(P)}$ at nominal value and at $V_{DD}=20$ V.

**Figure 12:**
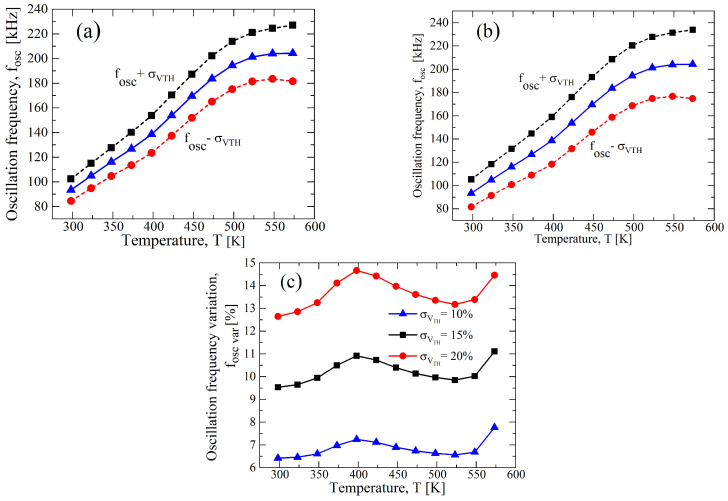
Results of a 1000 points process Monte Carlo analysis for (**a**) σ/μ=±0.15 and (**b**) σ/μ=±0.2 for only $V_{TH,N(P)}$, whereas $\mu_{N(P)}$ is constant at nominal value of Table 1 at $V_{DD}=20$ V. (**c**) $f_{OSC,var}$ as function of the temperature for different values of σ of $V_{TH,N(P)}$ with $\mu_{N(P)}$ at nominal value and at $V_{DD}=20$ V.

To stress the effect of process variations on the circuit performance, we increase σ/μ to ±15% and ±20% for $V_{DD}=20$ V, this being the bias condition defined during the design specification (see Table 1). In Figure 10, the results for a σ/μ of both parameters indicate an expected increase in the divergence from the nominal value, and observing Figure 10c, the percentage of variation of $f_{OSC}$ from the nominal value decreases with the temperature, but it has a peak at around T=400 K and a further increase over 500 K. To understand the behavior, we separately analyzed the σ/μ of the parameters, and the results are reported in Figure 11 and in Figure 12, respectively, for $\mu_{N(P)}$ and $V_{TH,N(P)}$. It is clear that the variation reduces for the $\mu_{N(P)}$-case with the increase in the temperature, whereas the $V_{TH,N(P)}$-case is almost constant, except for a maximum at T=400 K, which is related to that of Figure 10c. Moreover, both cases have an increase for temperatures that are higher than 500 K. In Table 5, we summarized the maximum variation for $f_{OSC}$, and the greater effect of the process variations for the $V_{TH,N(P)}$-case compared to the $\mu_{N(P)}$-case is clear.

**Figure 13:**
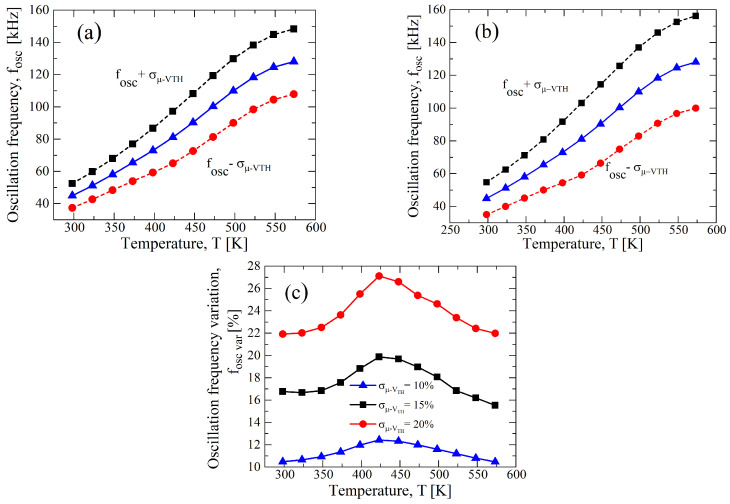
Results of a 1000 points process Monte Carlo analysis for (**a**) σ/μ=±0.15 and (**b**) σ/μ=±0.2 for $\mu_{N(P)}$ and $V_{TH,N(P)}$ at $V_{DD}=15$ V. (**c**) $f_{OSC,var}$ as function of the temperature for different values of σ of $\mu_{N(P)}$ and $V_{TH,N(P)}$ at $V_{DD}=15$ V.

**Figure 14:**
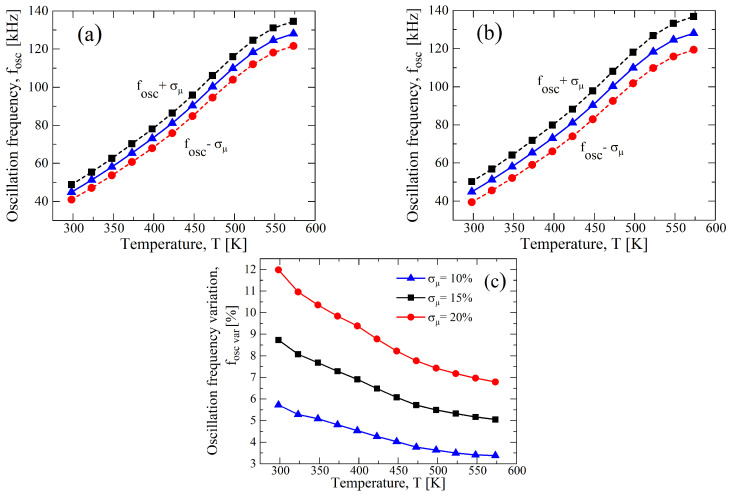
Results of a 1000 points process Monte Carlo analysis for (**a**) σ/μ=±0.15 and (**b**) σ/μ=±0.2 for only $\mu_{N(P)}$, whereas $V_{TH,N(P)}$ is constant at nominal value of Table 1 at $V_{DD}=15$ V. (**c**) $f_{OSC,var}$ as function of the temperature for different values of σ of $\mu_{N(P)}$ with $V_{TH,N(P)}$ at nominal value and at $V_{DD}=15$ V.

**Figure 15:**
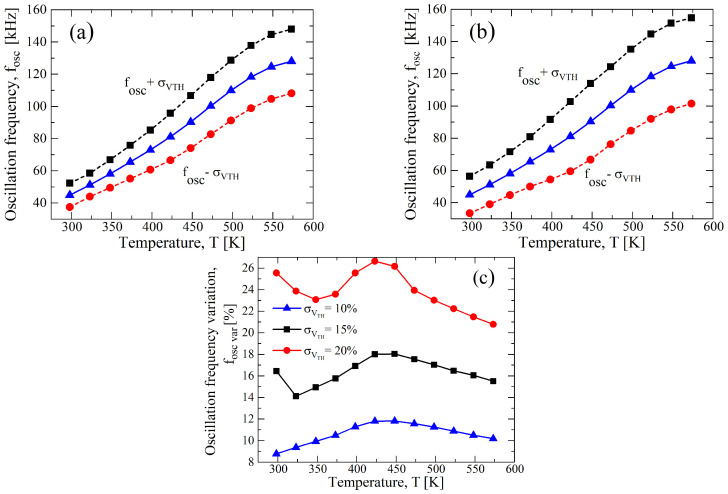
Results of a 1000 points process Monte Carlo analysis for (**a**) σ/μ=±0.15 and (**b**) σ/μ=±0.2 for only $V_{TH,N(P)}$, whereas $\mu_{N(P)}$ is constant at nominal value of Table 1 at $V_{DD}=15$ V. (**c**) $f_{OSC,var}$ as function of the temperature for different values of σ of $V_{TH,N(P)}$ with $\mu_{N(P)}$ at nominal value and at $V_{DD}=15$ V.

Considering that the best trade-off between linearity and circuit specification is at $V_{DD}=15$ V, we also performed Monte Carlo analysis for this case by increasing the σ/μ, and in Figure 13, the results are reported. Observing the curves, the 15 V-case has an increased variation compared with $V_{DD}=20$ V, which is more evident for T>400 K. It can be summarized through the $f_{OSC,var}$-*T* curve of Figure 13c, with a maximum variation of 27.11% at 425 K for a σ/μ=0.2. Instead, the deviation reduces in the high temperature range, contrary to the 20 V-case. Moreover, the separation of the effects of the process variation of $\mu_{N(P)}$ and $V_{TH,N(P)}$ have been analyzed and shown, respectively, in Figure 14 and in Figure 15: the prominent effect of $V_{TH,N(P)}$ is evident with respect to $\mu_{N(P)}$, which has at least double the value and defines the behavior of Figure 13c. It is interesting to note that the increase of the $f_{OSC,var}$ at T>500 K for $V_{DD}=20$ V disappears for the case of $V_{DD}=15$ V. In Table 5, we also report the maximum values of $f_{OSC,var}$ at $V_{DD}=15$ V, and higher values are shown than the 20V-case, so that although the 15V-case has a higher linearity, it is more greatly affected by process variations.

### 3.3. Results after Sensor Calibrations

If, on one hand, the bias voltage of $V_{DD}=20$ V reduces the dependency on the fabrication process of the oscillation frequency, on the other hand, the $f_{OSC}$-*T* characteristic has a scarce linearity. To overcome it, (16) suggests that it is possible to apply the one-point calibration procedure [16], where $f_{OSC}$-*T* curve is normalized by a $f_{OSC,1point}$ selected at a fixed temperature, $T_{1point}$, as follows:

(19) $$f_{OSC,norm}\left(T\right)=\frac{f_{OSC}\left(T\right)}{f_{OSC}\left(T_{1point}\right)}=\frac{f_{OSC}\left(T\right)}{f_{OSC,1point}}$$

**Figure 16:**
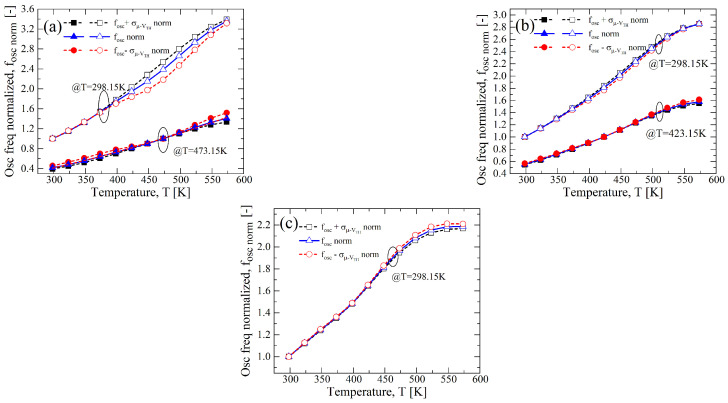
$f_{OSC,norm}$-*T* curves from the results of 1000 points Monte Carlo analysis with σ/μ=±0.1 for $V_{TH,N(P)}$ and $\mu_{N(P)}$. The nominal temperature and bias voltages values are, respectively, (**a**) $V_{DD}=12.5$ V, T=298.15 K, and 473.15 K; (**b**) $V_{DD}=15$ V, T=298.15 K, and 423.15 K; and (**c**) $V_{DD}=20$ V, T=298.15 K.

In this way, the negative effect of the process variation can be partially eliminated. Indeed, we applied it in the case of σ/μ=±0.1, both for $V_{TH,N(P)}$ and for $\mu_{N(P)}$, and in Figure 16, the results are shown for different bias conditions and $T_{1point}$. For $V_{DD}=12.5$ V of Figure 16a, a better correction is for $T_{1point}=473$ K compared to the room temperature case, as well as for $V_{DD}=15$ V of Figure 16b, where $T_{1point}=423$ K; instead, for $V_{DD}=20$ V, a $T_{1point}=298$ K can be used (see Figure 16c). This last result makes the bias supply of $V_{DD}=20$ V advantageous because an easier calibration procedure is applicable. Indeed, in term of the calibration easiness, the normalization at $T_{1point}=298$ K is of great advantage, but in the case of $V_{DD}=12.5$ V, it has a maximum variation of almost 8.65% from the nominal $f_{OSC,norm}$; instead, the same case evaluated at $T_{1point}=423$ K has a maximum variation of 5.93%.

**Figure 17:**
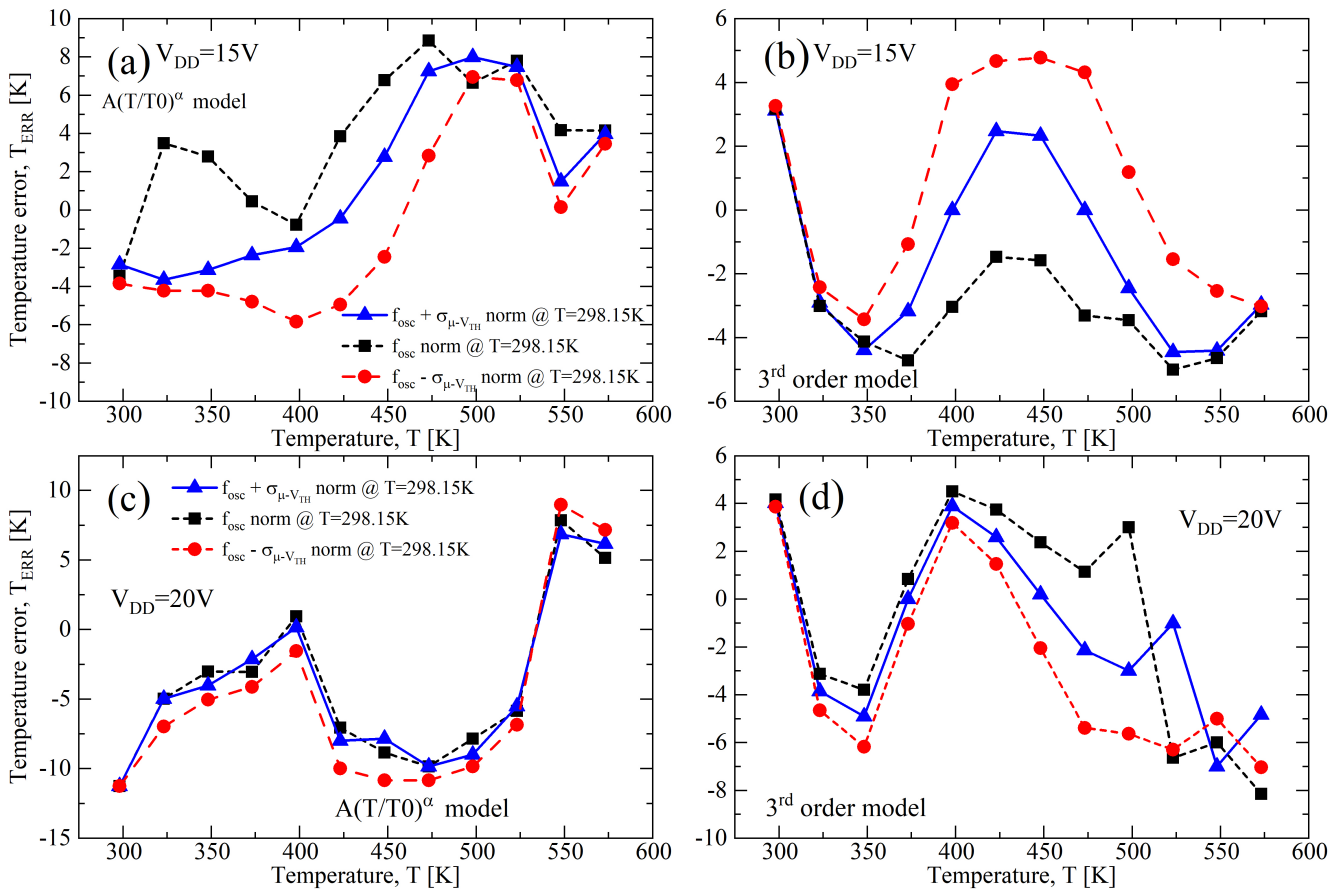
Temperature error as function of the temperature of the $f_{OSC,norm}$ in Figure 16 with σ/μ=±0.1 for $V_{TH,N(P)}$ and $\mu_{N(P)}$. The error is with respect to (16) at $V_{DD}$ (**a**) 15 V and (**c**) 20 V, and to a third-order curve model at $V_{DD}$ (**b**) 15 V and (**d**) 20 V.

Finally, once the calibration has been done, one can extract temperature from (16) using the measured frequency as the input variable. Hence, for the case of $V_{DD}=15$ V, one can use the parameters A=1.02 and α=1.64, and the calibration at $T_{1point}=298$ K reported in Figure 16b. In these conditions, the error between the extracted and effective temperatures, $T_{ERR}$, is shown in Figure 17a, and its maximum absolute value is 8.8 K across the whole temperature range between 298 K and 573 K. Furthermore, the process variations of σ/μ=±0.1, singularly either for $V_{TH,N(P)}$ or for $\mu_{N(P)}$, give errors of 8.8 K and 6.58 K, respectively. For the 20 V-case of Figure 17c, a maximum $T_{ERR}$ of −11.25 K has been found, both for the nominal case and for a process variation of σ/μ=±0.1. The error $T_{ERR}$ can be reduced if a third-order curve is used as model [16], and after a similar analysis, we obtained the results reported in Figure 17b for $V_{DD}=15$ V and in Figure 17d for $V_{DD}=20$ V, where $T_{ERR}$ reduces, respectively, at −5 K/4.78 K and −8.15 K/4.49 K.

### 3.4. Comparisons with the State-of-the-Art

An exhaustive comparison between the state-of-the-art and our proposal is difficult due to the limited availability of 4H-SiC temperature-frequency converter sensors. However, in Table 6, we report sensors based on similar operating principles and on other technology in order to understand how our proposal improves the state-of-the-art. The circuit of [16] is a Silicon CMOS technology circuit based on delay-locked loops and it shows an error of between +4 K and −4 K, which is slightly lower than ours, but in a narrower temperature range, i.e., [273.15;373.15] K. Indeed, as an example from a comparison with [28], our proposal has a greater temperature error, i.e., around 10%, but it can operate within a wider temperature range, even to 200 K, thanks to the higher performance of 4H-SiC technology compared to the Silicon one. Then, the proposals of [29,30], based on an Si 65nm-CMOS technology, use an area that is lower than ours, and in particular, [29] have −83% power dissipation and −66% temperature error, whereas [30] has, respectively, −44% and −83% compared to ours. Anyway, the dynamic power can be justified with the reduced channel length; instead, the error is lower because both the temperature variations of the Si transistor parameters are reduced due to the better $Si/SiO_{2}$ interface quality, and the temperature range is only 100 K compared to the value of 275 K for our sensor. In order to stress the comparison, we reduced the temperature range between 298 K and 443 K, extracting a $T_{ERR}$ of −3.15 K/4.58 K, which reduces to −3 K/3.84 K if a third-order model is used.

In Table 6, we report time-based temperature sensors fabricated using Silicon-On-Insulator technology. Ref. [31] is a Full-Depleted SOI at 28 nm CMOS and it has a very low $T_{ERR}$ as well as power consumption, and similarly, the SOI 32nm CMOS temperature sensors of [32] have a $T_{ERR}=\pm 1.95$ K. However, in both cases, although SOI-CMOS is much more mature technology than the 4H-SiC CMOS one, the temperature range is up to 385 K, which is 188 K lower than our proposal.

## 4. Layout

**Figure 18:**
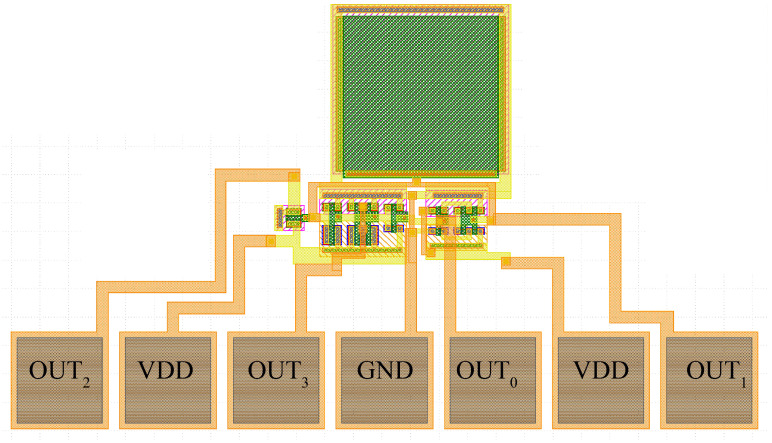
Layout of the proposed sensor.

The design results of Table 3 are used to draw the final layout of the circuit reported in Figure 18 with the Cadence Virtuoso 6.1.8 Layout software. The 4H-SiC 2 μm-CMOS process has 14 masks and two metal layers, whereas the active area of the sensor of Figure 1 is 0.163 mm${}^{2}$.

## 5. Conclusions

In this paper, the design of a 4H-SiC CMOS temperature sensor based on an analog oscillator is presented, as well as an analysis of its performances in terms of the process fabrication variations and the bias voltage. Unlike Silicon technology, our circuit showed a reduction in the propagation delay with an increase in the temperature, because the channel mobility improves at high temperature, and consequently, the frequency increases. The relation between the oscillation frequency and the temperature is almost linear, with an $R^{2}=0.9992$ at $V_{DD}=12.5$ V, but with a smaller influence of the process fabrication variation, i.e., 17.4% for $\sigma_{/}\mu =\pm 20\%$, is at $V_{DD}=20$ V, with an $R^{2}=0.9681$. On the other hand, performing a one-point calibration, we obtain a temperature error of +8.8 K and −5.8 K when $V_{DD}=15$ V.

The use of a simple model for the MOSFET current results are useful for a first-order approximation of the circuit design, but the effects of a high defects density at the $SiO_{2}/4H-SiC$ interface should be considered for a better description of the circuit. It means that a more accurate model has to be developed and the $\mu_{N(P)}$-dependency on the temperature should be focused on.

## Figures and Tables

**Table 1:** Circuit specifications.

Parameter	Unit	Value
$V_{DD}$	[V]	20
$V_{S}$	[V]	−8
$f_{OSC}$	[kHz]	90
$\frac{W_{N_{CAP}}}{L_{N_{CAP}}}$	$\left[\frac{\mu m}{\mu m}\right]$	$\frac{270}{270}$
$C_{1}$	[pF]	8
$C_{2}$	[pF]	8
$L_{min}$	[μm]	6
$V_{T^{+}}$	[V]	10
$V_{T^{-}}$	[V]	5

**Table 2:** Results of the analytical design of the proposed circuit using (1)–(14).

Parameter	Device	Value	Unit
$\frac{W}{L}$	$M_{N_{1}}$	36/6	$\left[\frac{\mu m}{\mu m}\right]$
	$M_{N_{2}}$	6/6	
	$M_{N_{3}}$	6/6	
	$M_{P_{1}}$	6/6	
	$M_{P_{2}}$	6/6	
	$M_{P_{3}}$	12/6	
	$M_{N_{4}}$	6/6	
	$M_{P_{4}}$	6/6	
	$M_{N_{5}}$	6/6	
	$M_{P_{5}}$	12/6	
	$M_{N_{6}}$	12/6	
	$M_{P_{6}}$	42/6	
	$M_{N_{7}}$	18/6	
$R_{S}$		94	[KΩ]

**Table 3:** Results of the design after the tuning using numerical simulations. The changed parameters compared to Table 2 are highlighted in red-bold.

Parameter	Device	Value	Unit
$\frac{W}{L}$	$M_{N_{1}}$	***6/6***	$\left[\frac{\mu m}{\mu m}\right]$
	$M_{N_{2}}$	6/6	
	$M_{N_{3}}$	6/6	
	$M_{P_{1}}$	6/6	
	$M_{P_{2}}$	6/6	
	$M_{P_{3}}$	12/6	
	$M_{N_{4}}$	6/6	
	$M_{P_{4}}$	6/6	
	$M_{N_{5}}$	***12/6***	
	$M_{P_{5}}$	***32/6***	
	$M_{N_{6}}$	***24/6***	
	$M_{P_{6}}$	***64/6***	
	$M_{N_{7}}$	***24/6***	
$R_{S}$		***60***	[KΩ]

**Table 4:** Maximum $f_{OSC,var}$ for σ/μ=±0.1 and at various $V_{DD}$.

$V_{\mathrm{DD}}$	$f_{\mathrm{OSC},\mathrm{var}}$		
	${\sigma /\mu |}_{V_{\mathrm{TH},N(P)}}$	${\sigma /\mu |}_{\mu_{N(P)}}$	${\sigma /\mu |}_{V_{\mathrm{TH},N(P)}-\mu_{N(P)}}$
12.5 V	22.64%	5.86%	23.33%
15 V	11.76%	5.72%	12.41%
20 V	7.24%	5.98%	8.41%

**Table 5:** $f_{OSC,var}$ maximum value for different σ/μ and at $V_{DD}=20$ V.

	$V_{\mathrm{TH},N(P)}$			$\mu_{N(P)}$			$V_{\mathrm{TH},N(P)}-\mu_{N(P)}$		
σ/μ	±10%	±15%	±20%	±10%	±15%	±20%	±10%	±15%	±20%
$V_{DD}=15$ V	11.82%	18.04%	26.64%	5.72%	8.73%	11.99%	12.42%	19.86%	27.11%
$V_{DD}=20$ V	7.24%	10.92%	14.66%	5.98%	9.03%	12.36%	8.41%	12.77%	17.4%

**Table 6:** Comparison between our proposal and the state-of-the-art.

	Range	Error	Bias Volt. $V_{\mathrm{DD}}$	Power	Area	Tech.
	**[K]**	**[K]**	**[V]**	**[mW]**	**[mm_2_]**	
This work (Num.Sim.)	298/573	−5.8/8.8	15	0.89	0.163	4H-SiC 2 μm-CMOS
[16]	273/373	−4/4	1.2	1.2	0.12	Si 130 nm-CMOS
[28]	233/378	± 0.5 (3σ)	5	2.5	2.3	Si 0.7 μm-CMOS
[29]	273/373	−3/3	1	0.154	0.004	Si 65 nm-CMOS
[30]	273/383	−1.5/1.5	1	0.5	0.008	Si 65 nm-CMOS
[31]	272/385	−2.5/1.2 ($V_{DD}$ = 0.6 V) −1.4/1.3 ($V_{DD}$ = 1.2 V)	0.6 V/1.2 V	0.056	0.001	FD-SOI 28 nm-CMOS
[32]	273/373	±1.95 (3σ)	1.65V	0.1	0.001	SOI 32 nm-CMOS

**Figure A1:**
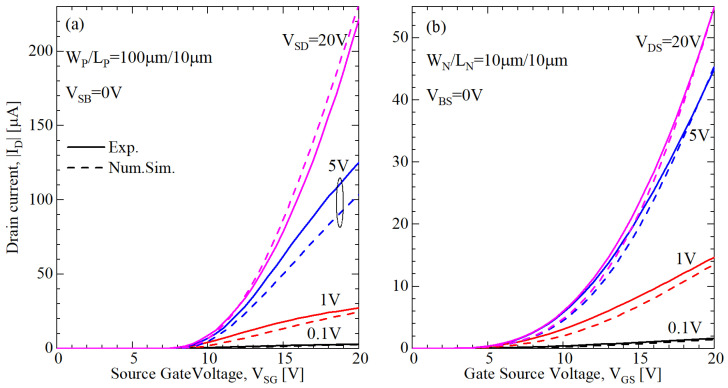
Comparison between the experimental and Verilog-A numerical model transcharacteristics of lateral 4H-SiC. (**a**) PMOSFET with $W_{P}/L_{P}=100$ μm/10 μm and (**b**) NMOSFET with $W_{N}/L_{N}=10$ μm/10 μm. All the curves are at r.t., $|V_{DS}|$ from 0.1 V to 20 V, and $|V_{BS}|=0$ V.

**Figure A2:**
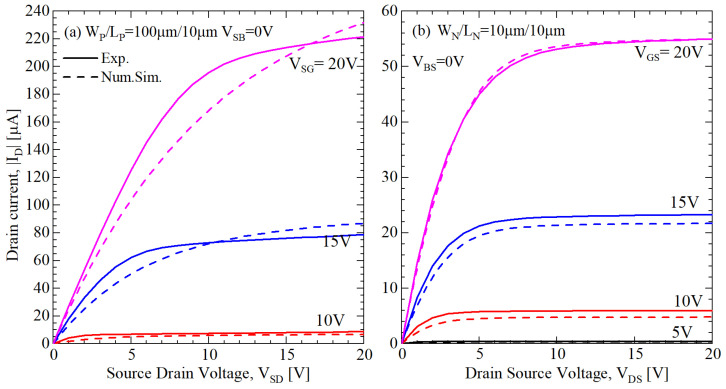
Comparison between experimental and Verilog-A numerical model output characteristics of lateral 4H-SiC. (**a**) PMOSFET with $W_{P}/L_{P}=100$ μm/10 μm and (**b**) NMOSFET with $W_{P}/L_{P}=10$ μm/10 μm. All the curves are at r.t. and $|V_{BS}|=0$ V.

**Figure A4:**
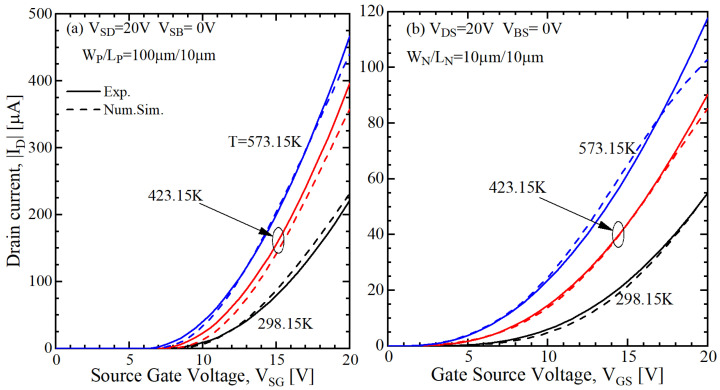
Comparison between experimental and Verilog-A numerical model transcharacteristics of lateral 4H-SiC. (**a**) PMOSFET with $W_{P}/L_{P}=100$ μm/10 μm and (**b**) NMOSFET with $W_{N}/L_{N}=10$ μm/10 μm at $|V_{DS}|=20$ V, $|V_{BS}|=0$ V, and for temperature, from 298 K to 573 K.

## Data Availability

A numerical simulation model has been developed and provided by Fraunhofer IISB. Please contact mathias.rommel@iisb.fraunhofer.de for further information.

## Appendix A

4H-SiC 2 μm-CMOS technology has been developed by Fraunhofer IISB (Germany), and the main physical parameters and the electrical parameters evaluated at room temperature are reported in Table A1.

**Table A1:** Physical and electrical parameters of the 4H-SiC CMOS technology at r.t.

Parameter	Unit	Value
$t_{OX}$	[nm]	55
$\epsilon_{OX}$	[fFcm${}^{-1}$]	345.31
$\epsilon_{SiC}$	[fFcm${}^{-1}$]	855.30
$C_{OX}$	[nFcm${}^{-2}$]	62.78
$V_{TH_{N}}$	[V]	5.8
$V_{TH_{P}}$	[V]	−8
$\mu_{N}$	[cm${}^{2}$V${}^{-1}$s${}^{-1}$]	17.14
$\mu_{P}$	[cm${}^{2}$V${}^{-1}$s${}^{-1}$]	3.52

The experimental transcharacteristics of Figure A1 and the output characteristics of Figure A2 are for PMOSFET and NMOSFET, with a form factor of 100 μm/10 μm and 10 μm/10 μm, respectively.

The curves of Figure A1 at $|V_{DS}|=100$ mV are used to extract both the threshold voltage, $V_{TH}$, and the mobility, $\mu_{N(P)}$. By using the second derivative method [33], we extracted the threshold voltages reported in Table A1, whereas the channel mobility dependency on the $V_{GS}$ is obtained using the following equation:

(A1) $$\mu =\frac{\frac{\partial I_{DS}}{\partial V_{GS}}{|}_{V_{DS}=100\;\mathrm{mV}}}{\frac{W}{L}C_{ox}V_{DS}}$$

and are reported in Figure A3a,b for PMOSFET and NMOSFET, respectively. Their mean value is the channel mobility reported in Table A1 and it is used to design the proposed circuit in Section 2. In Figure A3b, the unusual behavior of the NMOSFET channel mobility as a function of $V_{GS}$ is shown, and it has been described in Section 3 in order to justify the inconsistency between the expected specification and the numerical simulation results of the design in Table 2. Indeed, only PMOSFET channel mobility (see Figure A3a) shows typical step-like behavior, as well as a decrease with the increase in $V_{GS}$; instead, $\mu_{N}$ has a continuous increase until it reaches a maximum at around $V_{GS}=15$ V.

The temperature effect on the transistor currents are reported in Figure A4 for a $|V_{DS}|=20$ V, and it is clear that the increase in the current with the temperature occurs both for PMOSFET and NMOSFET. Such a behavior is due to a combination of phenomena: on one hand, the channel mobility increases up to 400 K and then decreases, as reported in [34] for a 4H-SiC lateral NMOS with a bulk doped $N_{A}=5\times 10^{17}$ cm${}^{-3}$; on the other hand, the threshold voltage continuously decreases with the temperature [35]. In order to take into account such behavior, a temperature dependency of $V_{TH}$ can be modeled with the following linear function [24]:

(A2) $$V_{TH}\left(T_{0}\right)\approx V_{TH}\left(T_{0}\right)\left[1+\beta \left(T-T_{0}\right)\right]$$

where β is a negative constant and we can neglect it from our analysis in a first approximation, because it is of the order of mV/K; on the other side, the temperature dependency of the channel mobility is modeled as follows [24,34]:

(A3) $$\mu \approx \mu \left(T_{0}\right){\left(\frac{T}{T_{0}}\right)}^{\alpha }$$

where α is a positive fitting parameter and is higher than 1.

It is worth noting that the high density of defects at the $4H-SiC/SiO_{2}$ interface is the reason for the improvement in the MOSFETs’ performance with the increase in temperature [36], whereas 4H-SiC bipolar devices showed worst behaviors under high-temperature operations; in particular, a thermal runaway of the current is observed [37,38], limiting their applications under harsh environment conditions.

The numerical simulations performed in Cadence Virtuoso are based on the compact BSIM4SiC model [22] and they have been opportunely developed by Fraunhofer IISB for the IISB’s 2 μm 4H-SiC CMOS technology. It is a extension of the widely used BSIM4 compact model, in which the typical physical phenomena of the 4H-SiC lateral n-channel and p-channel MOSFETs are considered. Indeed, the lower quality of the $4H-SiC/SiO_{2}$ interface induces a high density of interface trapped charges, differing significantly, in terms of the transfer and output characteristics, from the Silicon MOSFETs ones.

The effects include mobility degradation, a lower subthreshold slope, soft saturation, and an increase in the flat band and threshold voltages [22]. For an accurate description of the IISB’s 2 μm 4H-SiC CMOS technology, the parameters of the BSIM4SiC model have been determined through a parameter extraction procedure based on the measurement data of n- and p-type transistors. The developed compact model fits the experimental curves quite well, as shown in Figure A1 and Figure A2, with a higher accuracy for the NMOSFET case with respect to the PMOSFET case, especially for the output characteristics displayed in Figure A2a. The modeling of 4H-SiC PMOSFET is generally more challenging, due to the more limited knowledge about the defects at the $4H-SiC/SiO_{2}$ interface in the n-type body region with respect to 4H-SiC NMOSFET, which has had much more interest thanks to the power electronics applications. Then, the model also includes channel geometry scaling, which makes the parameter extraction and the achievement of satisfactory results even more complicated for all of the considered geometry. Moreover, in Figure A4, its validity is shown over a temperature range from 298 K to 573 K.
